# Editorial: Therapeutic targeting of cell death in cardiovascular diseases: from mechanisms to clinical applications

**DOI:** 10.3389/fcvm.2026.1885756

**Published:** 2026-09-03

**Authors:** Haiyan Xiang, Mohammed Ben Issa, Keman Xu, Shih-Yu Hung, Ying Shao, Yifan Lu, Bo Yuan, Juanjuan Liu, Fatma Saaoud, Shafqat Ullah, Juncheng Wei, Rihab Bouchareb, Liya Yin, Hong Wang, Xiaofeng Yang

**Affiliations:** 1 Center for Metabolic Disease Research and Sol Sherry Thrombosis Research Center, Department of Cardiovascular Sciences, Lewis Katz School of Medicine at Temple University, Philadelphia, PA, United States; 2 Lemole Center for Integrated Lymphatics and Vascular Research, Department of Cardiovascular Sciences, Lewis Katz School of Medicine at Temple University, Philadelphia, PA, United States; 3 College of Medicine Phoenix, University of Arizona, Phoenix, AZ, United States

**Keywords:** cardiovascular diseases, cell death, inflammation, precision medicine, therapeutics

Cardiovascular diseases (CVDs) remain the leading cause of morbidity and mortality worldwide despite substantial advances in prevention, diagnosis, and treatment. Increasing evidence indicates that regulated cell death (RCD) plays a fundamental role in cardiovascular injury, influencing inflammation, oxidative stress, metabolic remodeling, and tissue repair. As our understanding of these mechanisms has rapidly expanded, therapeutic targeting of regulated cell death has emerged as a promising strategy for limiting cardiovascular damage and improving clinical outcomes.

To highlight these advances, the Frontiers Research Topic “*Therapeutic Targeting of Cell Death in Cardiovascular Diseases: From Mechanisms to Clinical Applications*” was established to bring together original research and review articles that explore the molecular mechanisms of regulated cell death, their contribution to cardiovascular pathology, and their potential therapeutic applications. The seven articles included in this Research Topic collectively illustrate the remarkable evolution of the field, from mechanistic discovery and pathway characterization to translational approaches aimed at precision cardiovascular medicine. Together, these contributions emphasize that regulated cell death is no longer viewed simply as the terminal consequence of tissue injury but rather as a dynamic regulator of inflammation, metabolism, and tissue remodeling that represents an attractive therapeutic target.

Recent advances in cell death research and therapeutics have significantly expanded beyond the traditional apoptosis-centered paradigm (1). Newly recognized forms of programmed cell death, including ferroptosis, pyroptosis, necroptosis, cuproptosis, disulfidptosis, and the integrated cell death program PANoptosis, are increasingly implicated in tissue injury, inflammatory propagation, and repair failure (Table 1) (2–9). Among these pathways, ferroptosis has attracted particular attention because its dependence on iron metabolism and lipid peroxidation closely matches the metabolic and oxidative characteristics of cardiovascular tissues. Importantly, the field is shifting from asking simply whether cell death occurs to understanding when, where, and in which cell types distinct forms of regulated cell death are activated during disease progression.

**Table 1:** Programmed cell death and cardiovascular diseases.

Cell death type	Major regulators	Major features	Cardiovascular relevance
Apoptosis	Caspase-3, Bcl-2, Bax	Non-inflammatory programmed death	MI, HF
Pyroptosis	NLRP3, Caspase-1, GSDMD	Inflammatory pore formation	Atherosclerosis, MI
Ferroptosis	GPX4, ACSL4, iron	Lipid peroxidation	I/R injury, diabetic cardiomyopathy
Necroptosis	RIPK1, RIPK3, MLKL	Membrane rupture	HF, MI
Cuproptosis	Copper-TCA proteins	Copper toxicity	Emerging
Disulfidptosis	SLC7A11, NADPH	Cytoskeleton collapse	Emerging endothelial injury
PANoptosis	Pyroptosis + apoptosis + necroptosis	Integrated inflammatory death	Systemic inflammation

Emerging technologies, including single-cell sequencing, spatial transcriptomics, spatial metabolomics, and multi-omics integration, have enabled investigators to uncover organelle-specific, cell-specific, and stage-specific heterogeneity of regulated cell death. In parallel, increasing evidence demonstrates extensive crosstalk among oxidative stress, Ca^2^⁺ signaling, endoplasmic reticulum stress, mitochondrial dysfunction, inflammation, and metabolic remodeling, positioning regulated cell death as a dynamic systems-level process rather than an isolated biological event. Therapeutically, iron chelators, lipid peroxidation inhibitors, glutathione peroxidase 4 (GPX4) stabilizers, NFE2 like BZIP transcription factor 2 (NFE2L2, Nrf2) activators, mitochondrial protectors, and natural compounds have all shown promising potential in preclinical studies. Consequently, the future development of cell death therapeutics will likely depend on precise temporal intervention, cell-specific targeting, biomarker-guided therapy, and integrated modulation of metabolism and immune signaling (10).

In this editorial, we summarize the major advances presented in this Research Topic, discuss emerging concepts in regulated cell death biology, and highlight how these studies collectively advance our understanding of cardiovascular disease while pointing toward future opportunities for precision therapeutic intervention (Table 2).

**Table 2:** Summary of the studies included in this research topic.

Author	Paper Title	Primary cell death pathway	Main idea	PMID/DOI
Cao et al.	*Ferroptosis: key regulatory pathways and their implications in cardiovascular pathophysiology*	Ferroptosis	Ferroptosis is an iron-dependent regulated cell death mechanism driven by lipid peroxidation; it contributes to cardiovascular damage, inflammation, and remodeling.	DOI: 10.3389/fcvm.2026.1732010
Carlson et al.	THR-123, a novel BMP-7 mimetic that activates AKT phosphorylation and inhibits cardiomyocyte apoptosis and inflammation, protects heart from myocardial injury (MI) in rat model(eary time anti-apoptosis)	Apoptosis/inflammation-related injury	THR-123 activates AKT phosphorylation and reduces cardiomyocyte apoptosis and inflammation in a myocardial injury model.	DOI: 10.3389/fcvm.2026.1729074
Chen et al.	The potential therapeutic value of the natural plant compounds matrine and oxymatrine in cardiovascular diseases	Multiple/indirect cell death-related protection	Matrine and oxymatrine exert cardioprotective effects through anti-inflammatory, anti-oxidative, anti-atherosclerotic, anti-remodeling, and multi-pathway regulation.	PMID: 39041001
Pang et al.	Temporal regulation of genetic programs governing multiple cell death during myocardial ischemia-reperfusion injury	Multiple: apoptosis, ferroptosis, necrosis, pyroptosis, autophagy, cuproptosis	Different cell death programs dominate at different reperfusion stages; apoptosis occurs early, while ferroptosis, necrosis, and pyroptosis become important during prolonged reperfusion.	PMID: 40979591
Wang et al.	Bioinformatics analysis and experimental validation of ferroptosis genes in heart failure and atrial fibrillation	Ferroptosis	Identifies ferroptosis-related genes including TFRC, CP, SAT1, STEAP3, AKR1C1, and LPCAT3 as potential shared biomarkers and targets for HF and AF.	PMID: 40672390
Zhang et al.	Tanshinone I promotes angiogenesis and improves ventricular remodeling post-myocardial infarction via ALDH2 signaling-mediated ferroptosis inhibition	Ferroptosis	Tanshinone I activates ALDH2 signaling, inhibits endothelial cell ferroptosis, promotes angiogenesis, and improves ventricular remodeling after MI.	PMID: 40642000
Zhang et al.	Transient receptor potential channels as key regulators of cell death in atherosclerosis	Multiple:apoptosis, autophagy, necrosis; potential ferroptosis connection	TRP channels regulate Ca2 + influx, oxidative stress, inflammation, and multiple cell death pathways in atherosclerosis.	DOI: 10.3389/fimmu.2025.1661805

## Therapeutic targeting of regulated cell death in cardiovascular disease: toward precision inflammatory targeting

The studies included in this Research Topic reinforce the emerging concept that cardiovascular diseases (CVDs) are not merely disorders of hemodynamics or vascular obstruction but are also chronic inflammatory and metabolic diseases driven by multiple forms of regulated cell death (RCD). Rather than representing isolated terminal events, these pathways are now recognized as dynamic regulators of inflammation, redox homeostasis, mitochondrial integrity, cytoskeletal organization, and intercellular communication. Consequently, the therapeutic value of targeting regulated cell death extends beyond preventing cellular loss to interrupting inflammatory propagation, preserving tissue homeostasis, and limiting adverse cardiac and vascular remodeling.

Among the emerging RCD pathways, pyroptosis has become one of the most prominent inflammatory mechanisms implicated in cardiovascular disease. Pyroptosis is initiated by inflammasome activation, particularly through assembly of the NLR family pyrin domain containing 3 (NLRP3) inflammasome, which activates caspase-1 and promotes cleavage of gasdermin D (GSDMD). The pore-forming N-terminal fragment of GSDMD disrupts plasma membrane integrity, resulting in membrane rupture and the release of the pro-inflammatory cytokines interleukin-1β (IL-1β) and IL-18. Beyond these well-established mechanisms, recent evidence suggests that pyroptotic macrophages release extracellular vesicles (EVs) containing functional GSDMD pores that can propagate inflammatory injury to neighboring endothelial cells, vascular smooth muscle cells (VSMCs), and macrophages. This proposed EV-GSDMD propagation model expands the traditional view of pyroptosis from a cell-autonomous death pathway to an important mechanism of intercellular inflammatory amplification. Within atherosclerotic lesions, this process may contribute to plaque instability, necrotic core expansion, and diffuse vascular inflammation, thereby perpetuating disease progression through sterile inflammatory signaling.

Ferroptosis represents another major advance in cardiovascular cell death biology and has emerged as one of the most promising therapeutic targets because of its close association with metabolic dysfunction and oxidative stress. Unlike apoptosis, ferroptosis is a metabolism- and redox-dependent form of regulated cell death characterized by iron-dependent lipid peroxidation. Excessive reactive oxygen species (ROS), glutathione depletion, GPX4 dysfunction, and mitochondrial oxidative stress collectively promote catastrophic membrane lipid damage and cellular dysfunction. Ferroptosis appears particularly relevant to ischemia-reperfusion injury, diabetic cardiomyopathy, endothelial dysfunction, and heart failure, conditions that share common features including mitochondrial instability, nicotinamide adenine dinucleotide phosphate (NADPH) depletion, and oxidative injury. During myocardial reperfusion, the restoration of oxygen paradoxically generates bursts of ROS while simultaneously mobilizing intracellular iron, creating conditions that strongly favor ferroptotic injury. Consequently, ferroptosis represents a mechanistic link between mitochondrial dysfunction, metabolic remodeling, and cardiovascular disease progression.

The recently described pathway of disulfidptosis further expands the conceptual relationship between metabolism and regulated cell death. Under conditions of NADPH depletion, excessive cystine uptake through solute carrier family 7 member 11 (SLC7A11) promotes intracellular disulfide accumulation, leading to collapse of the actin cytoskeleton. In contrast to ferroptosis, which is driven primarily by lipid peroxidation, disulfidptosis identifies cytoskeletal integrity as a critical sensor of metabolic stress. This mechanism may be particularly relevant to endothelial biology because maintenance of endothelial barrier function depends heavily on cytoskeletal organization. Conditions such as diabetes, sepsis, and ischemia-reperfusion injury may therefore induce a disulfidptosis-like endothelial phenotype characterized by vascular leakage, microvascular dysfunction, and inflammatory cell infiltration.

Increasing evidence also supports the concept of PANoptosis, an integrated inflammatory cell death program involving coordinated activation of pyroptosis, apoptosis, and necroptosis. This framework helps explain why cardiovascular injury rarely results from activation of a single death pathway. Instead, myocardial infarction, heart failure, and sepsis-associated cardiomyopathy frequently involve simultaneous activation of multiple inflammatory death mechanisms that amplify one another through damage-associated molecular pattern (DAMP) release, cytokine signaling, mitochondrial dysfunction, and oxidative stress. Recognition of this extensive crosstalk suggests that selective inhibition of one pathway alone may inadvertently redirect cells toward alternative inflammatory death programs, highlighting both the complexity and the therapeutic challenges of targeting regulated cell death in cardiovascular disease.

Mechanistic advances in regulated cell death have stimulated the development of several promising therapeutic strategies. Clinically validated anti-inflammatory approaches include inhibition of IL-1β signaling with canakinumab and anakinra. Notably, the CANTOS trial demonstrated that IL-1β blockade significantly reduced recurrent cardiovascular events independently of lipid lowering, providing compelling clinical evidence that inflammatory cell death contributes directly to cardiovascular disease progression. Additional therapies with indirect anti-inflammatory or cytoprotective effects, including statins, sodium-glucose cotransporter-2 (SGLT2) inhibitors, glucagon-like peptide-1 (GLP-1) receptor agonists, and colchicine, further support the concept that modulation of inflammatory signaling can improve cardiovascular outcomes. At the experimental level, emerging therapies include NLRP3 inhibitors such as MCC950, GSDMD inhibitors including disulfiram, receptor interacting serine/threonine kinase 1 (RIPK1) inhibitors targeting necroptosis, and ferroptosis inhibitors such as Ferrostatin-1 and Liproxstatin-1. Although these approaches have shown considerable promise in preclinical models, their clinical translation remains challenging because regulated cell death pathways exhibit substantial tissue specificity, temporal heterogeneity, and disease-context dependence.

Accordingly, the greatest challenge facing cardiovascular cell death therapeutics is not simply identifying effective molecular inhibitors but achieving translational precision. Cardiomyocytes are terminally differentiated and highly dependent on mitochondrial metabolism, endothelial cells require preservation of barrier integrity, and inflammatory responses differ markedly between sterile cardiovascular injury and infectious diseases. Moreover, prolonged inhibition of inflammatory cell death pathways may compromise host antimicrobial defense or induce compensatory activation of alternative death programs. Future therapeutic strategies will therefore require tissue-specific drug delivery, precise temporal intervention, biomarker-guided patient stratification, and a more comprehensive understanding of the dynamic interactions among regulated cell death pathways. As emphasized by the studies presented in this Research Topic, successful translation of cell death therapeutics into cardiovascular medicine will likely depend on integrated modulation of metabolism, inflammation, and immune signaling rather than inhibition of individual pathways in isolation.

## Highlights of the research topic: emerging cell death programs and mechanistic advances

The seven articles included in this Research Topic collectively illustrate the remarkable evolution of cardiovascular cell death research from descriptive characterization of individual death pathways toward a multidimensional, spatiotemporally regulated, and therapeutically actionable framework. Rather than functioning as isolated mechanisms of cellular demise, regulated cell death pathways are increasingly recognized as interconnected biological networks that integrate oxidative stress, metabolic remodeling, inflammation, vascular dysfunction, and tissue repair. Together, the studies in this collection provide complementary mechanistic and translational perspectives that substantially advance our understanding of cardiovascular disease.

A major theme emerging from this Research Topic is the central role of ferroptosis in cardiovascular pathology. Cao et al. comprehensively reviewed the molecular mechanisms governing ferroptosis, emphasizing the coordinated contributions of iron redistribution, lipid peroxidation, mitochondrial dysfunction, antioxidant defense systems, and organelle stress to cardiovascular injury. Their work highlights ferroptosis as more than a distinct form of regulated cell death; it represents a critical intersection between metabolic dysregulation, oxidative stress, and inflammatory signaling (8, 11). Complementing these mechanistic insights, Wang et al. identified shared ferroptosis-associated gene signatures linking heart failure and atrial fibrillation, suggesting that ferroptosis contributes not only to cardiomyocyte loss but also to electrical remodeling, structural remodeling, and disease progression across distinct cardiovascular disorders. Extending these findings to the vascular compartment, Zhang et al. demonstrated that endothelial ferroptosis impairs angiogenesis following myocardial infarction and proposed that suppression of ferroptosis through aldehyde dehydrogenase 2 family member (ALDH2)-dependent mechanisms promotes vascular repair and improves ventricular remodeling. Collectively, these studies establish ferroptosis as a central therapeutic target acting at multiple levels of cardiovascular pathophysiology, including myocardial injury, endothelial dysfunction, tissue repair, and adverse remodeling.

Another important concept emerging from this collection is the dynamic and context-dependent nature of regulated cell death (12). Pang et al. demonstrated that apoptosis, autophagy, ferroptosis, pyroptosis, and other regulated cell death pathways exhibit distinct temporal activation patterns during myocardial ischemia-reperfusion injury. Their findings challenge the traditional view that a single death mechanism predominates throughout disease progression and instead suggest that regulated cell death evolves continuously during injury and repair. This temporal heterogeneity has important therapeutic implications, indicating that effective interventions may require stage-specific modulation of different cell death programs rather than uniform inhibition of a single pathway throughout the course of disease (13).

Beyond ferroptosis, several contributions emphasize the extensive crosstalk between cellular signaling pathways that regulate cardiovascular injury. The review of transient receptor potential (TRP) channels highlights how calcium influx, mechanosensing, oxidative stress, and mitochondrial dysfunction converge to regulate multiple forms of programmed cell death during atherosclerosis. By linking ion homeostasis with inflammatory signaling, this work illustrates the complex molecular interactions that coordinate vascular injury and remodeling. Similarly, the review of matrine and oxymatrine expands the therapeutic landscape by demonstrating how naturally derived compounds exert cardioprotective effects through simultaneous modulation of oxidative stress, inflammation, mitochondrial function, and multiple regulated cell death pathways. These findings reinforce the concept that successful therapeutic strategies may benefit from targeting interconnected biological networks rather than individual molecular mediators (14, 15).

Taken together, the studies presented in this Research Topic demonstrate that regulated cell death has evolved from being viewed as a passive consequence of cardiovascular injury to an active regulatory network governing inflammation, metabolism, intercellular communication, angiogenesis, tissue remodeling, and cardiac repair. More importantly, they collectively support a paradigm shift toward precision cardiovascular medicine, in which therapeutic modulation of regulated cell death will likely require consideration of disease stage, cellular context, metabolic status, and the dynamic interactions among multiple death pathways. These advances provide a strong mechanistic foundation for developing next-generation therapies capable of interrupting inflammatory propagation while promoting tissue repair and functional recovery in cardiovascular disease.

## Conclusions and future directions

The studies assembled in this Research Topic illustrate the remarkable progress achieved in understanding regulated cell death in cardiovascular disease. Emerging mechanisms such as ferroptosis, pyroptosis, disulfidptosis, cuproptosis, and PANoptosis have expanded the traditional view of myocardial injury and revealed new opportunities for therapeutic intervention. At the same time, these pathways are highly interconnected, emphasizing the need for precise temporal, cell-specific, and disease-stage–dependent therapeutic strategies. Future integration of single-cell transcriptomics, spatial multi-omics, systems biology, and biomarker-guided clinical trials will facilitate translation of these mechanistic discoveries into precision cardiovascular medicine. Collectively, the articles in this Research Topic provide an important framework for advancing the next generation of cell death-targeted therapies.

## References

[1] Kerr JF Wyllie AH Currie AR. Apoptosis: a basic biological phenomenon with wide-ranging implications in tissue kinetics. Br J Cancer. (1972) 26(4):239–57. 10.1038/bjc.1972.33 4561027 PMC2008650

[2] Heinke L. The bystander effect of pyroptosis. Nat Rev Mol Cell Biol. (2025) 26(3):174. 10.1038/s41580-025-00831-6 39905194

[3] Wright SS Kumari P Fraile-Ágreda V Wang C Shivcharan S Kappelhoff S. Transplantation of gasdermin pores by extracellular vesicles propagates pyroptosis to bystander cells. Cell. (2025) 188(2):280–91.e17. 10.1016/j.cell.2024.11.018 39742811 PMC12272064

[4] Beltrán-Visiedo M Soler-Agesta R Sarosiek KA Green DR Galluzzi L. Regulation of inflammatory processes by caspases. Nat Rev Mol Cell Biol. (2025) 26(11):884–901. 10.1038/s41580-025-00869-6 40603684

[5] Gan B. Redox-driven cell death by disulfidptosis and its therapeutic potential. Nat Rev Mol Cell Biol. (2025) 26(10):727–9. 10.1038/s41580-025-00888-3 40847224

[6] Libby P. Inflammation in atherosclerosis. Nature. (2002) 420(6917):868–74. 10.1038/nature01323 12490960

[7] Tonnus W Linkermann A. The in vivo evidence for regulated necrosis. Immunol Rev. (2017) 277(1):128–49. 10.1111/imr.12551 28462528

[8] Stockwell BR. Ferroptosis turns 10: emerging mechanisms, physiological functions, and therapeutic applications. Cell. (2022) 185(14):2401–21. 10.1016/j.cell.2022.06.003 35803244 PMC9273022

[9] Galluzzi L Vitale I Aaronson SA Abrams JM Adam D Agostinis P. Molecular mechanisms of cell death: recommendations of the Nomenclature committee on cell death 2018. Cell Death Differ. (2018) 25(3):486–541. 10.1038/s41418-017-0012-4 29362479 PMC5864239

[10] Tang D Kang R Berghe TV Vandenabeele P Kroemer G. The molecular machinery of regulated cell death. Cell Res. (2019) 29(5):347–64. 10.1038/s41422-019-0164-5 30948788 PMC6796845

[11] Jiang X Stockwell BR Conrad M. Ferroptosis: mechanisms, biology and role in disease. Nat Rev Mol Cell Biol. (2021) 22(4):266–82. 10.1038/s41580-020-00324-8 33495651 PMC8142022

[12] Newton K Strasser A Kayagaki N Dixit VM. Cell death. Cell. (2024) 187(2):235–59. 10.1016/j.cell.2023.11.044 38242081

[13] Kayagaki N Webster JD Newton K. Control of cell death in health and disease. Annu Rev Pathol Mech Dis. (2024) 19:157–80. 10.1146/annurev-pathmechdis-051022-014433 37788577

[14] Qi K Mu Y Hu Y Li J Liu J. Comprehensive landscape of cell death mechanisms: from molecular cross-talk to therapeutic innovation in oncology. Front Cell Dev Biol. (2025) 13:1611055. 10.3389/fcell.2025.1611055 40741332 PMC12308763

[15] Wang J Lai B Nanayakkara G Yang Q Sun Y Lu Y. Experimental data-mining analyses reveal new roles of low-intensity ultrasound in differentiating cell death regulatome in cancer and non-cancer cells via potential modulation of chromatin long-range interactions. Front Oncol. (2019) 9:600. 10.3389/fonc.2019.00600 31355136 PMC6640725

